# Dynamic inflammatory response among routine laboratory biomarkers and their predictive ability for mortality in patients with severe COVID-19

**DOI:** 10.3389/fmed.2022.1047304

**Published:** 2022-11-17

**Authors:** Arturo Cortes-Telles, Ana Ligia Gutiérrez-Solis, Víctor Aarón Álvarez-Sánchez, Alejandro Gabriel González-Garay, Roberto Lugo, Azalia Avila-Nava

**Affiliations:** ^1^Clinica de Enfermedades Respiratorias, Hospital Regional de Alta Especialidad de la Península de Yucatán, Mérida, Mexico; ^2^Unidad de Investigación, Hospital Regional de Alta Especialidad de la Península de Yucatán, Mérida, Mexico; ^3^Departamento de Medicina Interna, Hospital Regional de Alta Especialidad de la Península de Yucatan, Mérida, Mexico; ^4^Departamento de Metodología de la Investigación, Instituto Nacional de Pediatría, Ciudad de México, Mexico

**Keywords:** SARS-CoV-2, Mexican population, leukocytes, neutrophils, CRP, D-dimer

## Abstract

**Background:**

The severity of coronavirus disease 2019 (COVID-19) is related to several factors, including age, sex, and comorbidities (obesity, type 2 diabetes, and hypertension). However, systemic inflammation plays a fundamental role in COVID-19 pathophysiology. Several studies have described this association employing specific biomarkers that are not routinely used in clinical practice. On the other hand, very few reports in the literature focused on the analysis of the routine laboratory biomarkers to predict the outcome of severe COVID-19 patients.

**Objective:**

We aimed to analyze the dynamic inflammatory response using routine laboratory biomarkers to predict in-hospital mortality in Mexican patients with severe COVID-19.

**Methods:**

This is a cohort study including patients with severe COVID-19. Demographic characteristics were retrieved from medical charts and biochemical parameters were measured at hospital admission and subsequently on days 3, 5, 7, 10, 14, and 21 during the hospital stay; measurements were stopped when patients were discharged from the hospital (alive or death).

**Results:**

A total of 250 patients were included in the study, 40.8% of patients died. The analyzed routine laboratory parameters, such as serum levels of neutrophil-to-lymphocyte ratio, C-reactive protein, and D-dimer remained elevated in hospitalized patients who did not survive, whereas eosinophil and platelets were maintained at lower levels. In the multivariate analysis, leukocytes, and neutrophils were the best biomarkers for predicting mortality risk and were independent of age, gender, or comorbidities.

**Conclusion:**

Our results support the use of routine laboratory biomarkers as predictors of mortality in Mexican hospitalized patients with severe COVID-19.

## Introduction

Coronavirus disease 2019 (COVID-19), caused by severe acute respiratory syndrome coronavirus 2 (SARS-CoV-2) infection, has been spreading worldwide with 625,740,449 confirmed cases of COVID-19, including 6.563.667 deaths by 26 October 2022 (1). COVID-19 disease is clinically heterogeneous, the most common symptoms are fever (55–65%), cough (45–50%) and respiratory distress (27.5%), whereas diarrhea (9.5%) and vomiting (6.5%) are less common (2). In severe cases, the disease can cause systemic inflammation, conditioning multiple organ failure, and the appearance of complications such as acute respiratory distress syndrome (ARDS), acute cardiac injury, acute kidney injury, acute liver injury, sepsis, shock, coagulopathy, and pulmonary embolism, that require hospitalization (3–5). Vaccination decreased the rate of hospitalized patients need to be transferred to the intensive care unit (ICU), moving from 20.6% to around 2% (6). Latin America became one of the most affected regions, and Mexico ranked as one of the first places regarding the number of deaths (7). This situation in Mexico was related to the high prevalence of different comorbidities and conditions such as obesity, high blood pressure (HBP), and type 2 diabetes (T2D). In fact, these factors increase the risk of death up to 7.7 times (8). Furthermore, the presence of these abnormalities is not the only factor in COVID-19 pathogenesis. These pathologies are related to an increased proinflammatory state, which plays a fundamental role in the evolution and severity of COVID-19 (9). This response enhances the activation of the immune system, promoting an increase in the neutrophil count with a substantial reduction of CD4(+) T cell and CD8(+) T cell counts (effector T cells) altogether with the release of proinflammatory markers such as C-reactive protein (CRP), D-dimer, and several cytokines (8–11). The secretion of proinflammatory cells leads to an aberrant inflammatory response, named “cytokine storm,” a potentially fatal immune disease, considered to be the main cause of disease severity and death in patients with COVID-19 (9, 12). There is little information about the role of inflammation on the severity and mortality of COVID-19 in the Mexican population. One study showed that serum biomarkers such as albumin (OR 3.76 [CI 95% 1.56–9.07], *P* = 0.003), lactate dehydrogenase (OR 5.45 [CI 95%2.36–12.57], *P* < 0.001) and neutrophil-to-lymphocyte (N/L) ratio (OR 4.64 [CI 95% 2.05–10.53], *P* < 0.001) were independent risk factors associated with mortality in COVID-19 patients (13). Another study showed that proinflammatory cytokine interlukin-6 (HR 1.01 [CI 95% 1.003–1.020], *P* < 0.011) is a risk factor associated with mortality in Mexican individuals (14). Nonetheless, information is limited in low-income countries, and further research is needed. Prognostic predictors obtained from routine laboratory biomarkers during the early phase of the disease might be useful to make timely treatment decisions; as well as predicting clinical progression and disease severity. The aim of the present study was to determine the dynamic inflammatory response among routine laboratory biomarkers and mortality in Mexican patients with severe COVID-19.

## Materials and methods

### Study design

A cohort study among patients attended in the COVID specialty unit between October 2020 and April 2021, whose fulfilled World Health Organization (WHO) diagnostic criteria for severe COVID-19 (15) and confirmed by reverse transcriptase-polymerase chain reaction (RT-PCR) for SARS-CoV-2. The study was approved by the Ethics Committee of the Hospital Regional de Alta Especialidad de la Península de Yucatán (2020-023), and it was conducted in accordance with the Declaration of Helsinki. Written informed consent was obtained from all participants. This study followed the Strengthening the Reporting of Observational Studies in Epidemiology (STROBE) reporting guideline (16).

### Study population

Selection criteria of patients were as follows, inclusion criteria included: clinical signs of pneumonia (fever, cough, dyspnea, fast breathing) and respiratory rate >30 breaths/min; severe respiratory distress or O_2_ saturation <90% on room air; meanwhile exclusion criteria were: patients diagnosed with critical COVID-19 according to WHO criteria (15) or any other alternative diagnosis: non-COVID-19 pneumonia, acute heart failure, myocardial infarction, acute kidney insufficiency, and hepatic insufficiency.

### Data collection: Clinical characteristics, blood sample collection, and routine laboratory biomarkers

Data were collected at admission, including sex, age, and self-reported comorbidities like HBP, T2D, and obesity (BMI > 30kg/m^2^). Moreover, clinical characteristics were documented, such as oxygen saturation (O2 saturation), among others. Blood samples were individually collected at hospital admission and every 72 h for 21 days. Measurements were stopped at any time if either of the following: (1) patients were discharge alive or (2) death in-hospital. Biochemical parameters such as complete blood count (CBC), CRP, D-dimer were included, and the N/L ratio was calculated. The concentration of CRP was measured by immunoturbidimetric test (CRPL3^®^), D-dimer was evaluated by automated latex enhanced immunoassay f (Hemosil^®^ D-dimer HS 500) and CBC by automatic flow cytometry using a semiconductor laser for leukocyte analysis (XT-2000i^®^).

### Statistical analyses

Continuous variables were evaluated using the Kolmogorov–Smirnov test to analyze the type of distribution. The patients were classified as groups of survivors or non-survivors. Data are presented as the mean ± standard deviation (SD) or medians and 95% confidence interval [CI 95%]. Categorical variables are reported as frequencies. Differences between groups were performed with the Mann–Whitney U test for quantitative variables. The proportions were analyzed through the chi-squared statistical test. The risk of death by COVID-19 was analyzed considering the behavior of the variables from the time the participants entered the hospital until their discharge. The cut-off values of routine laboratory biomarkers were determined, and survival analysis was performed using the Kaplan–Meier method. Random-effects parametric survival-time model analysis was performed and generated a model unadjusted and adjusted by sex, age, and comorbidities. Data from participants with at least two measurements were considered for inclusion in the analysis. A value of *P* < 0.05 was considered significant. Data were analyzed using Stata V15.1.

## Results

### General characteristics of the study population

**FIGURE 1:**
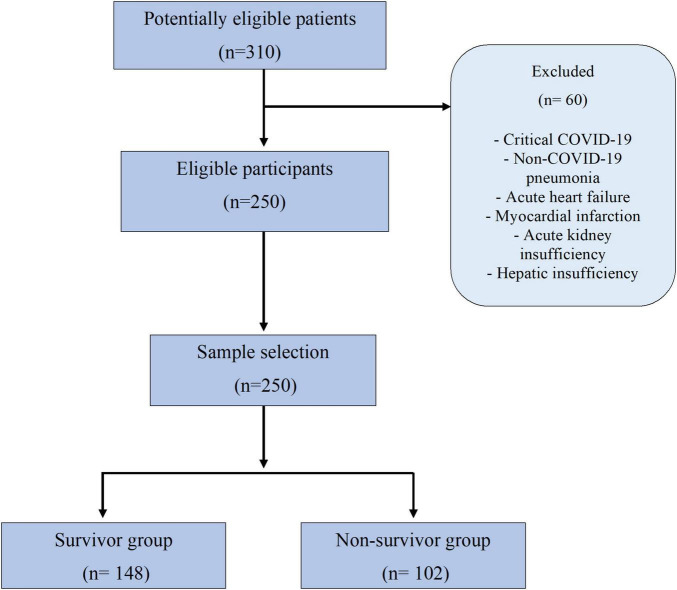
Flow chart.

A total of 250 patients were included in the study, the mean age was 54.3 ± 15.3 years, and 68% were male (Figure 1). Mean of period of hospitalization was 9.42 ± 8.23 days and the most frequent comorbidities in the population included were obesity (51.6%), followed by HBP (33.2%) and T2D (30.4%). Baseline levels of different routine laboratory biomarkers were included, highlighting eosinophils (0.05 ± 0.10 × 10^3^ cells/μl), CRP (174.63 ± 123.76 ng/ml), D-dimer (2010 ± 3584 ng/dl), and N/L ratio (11.9 ± 10.8) (Supplementary Table 1).

### Comparison of clinical characteristics between populations according to survival status

Overall, we registered a mortality of 40.8% (*n* = 102). The comparisons according to survival status are summarized in Table 1. The presence of comorbidities such as HBP, T2D, obesity, chronic obstructive pulmonary disease (COPD), cardiopathy, chronic kidney disease (CKD), hepatopathy, human immunodeficiency virus (HIV), and dyslipidemia did not show significant differences according to the survival status in the patients (Supplementary Table 2).

**TABLE 1:** Comparative analysis of routine biochemical parameters of population according to survival status (*n* = 250).

	Survivors *N* = 148	Non-Survivors *N* = 102	*P*-value
Leukocytes (×10^3^ cells/μL)	9.5 [7.3, 12.7]	13.1 [10.3, 17.8]	< 0.001
Neutrophils (×10^3^ cells/μL)	7.5 [7.3, 7.9]	11.9 [11.3,12.5]	< 0.001
Lymphocytes (×10^3^ cells/μL)	1.3 [1.2, 1.5]	0.9 [0.8, 1.0]	0.157
Eosinophil (×10^3^ cells/μL)	0.07 [0.02, 0.18]	0.02 [0.01, 0.08]	< 0.001
Platelets (×10^3^ cells/μL)	371 [350, 419.4]	291 [286.0, 293.5]	0.014
CRP (mg/mL)	76.5 [71.7, 81.6]	170 [150, 207]	< 0.001
D-dimer (ng/mL)	975 [757, 1516]	2035 [1600, 2517]	< 0.001
ERS (mm/h)	31 [26.5, 34]	33.7 [30.2, 40.2]	0.001
N/L ratio	5.63 [5.50, 6.11]	13.9 [12.2, 14.5]	< 0.001

Data are presented as median and Quartile 1, Quartile 3 [Q1, Q3]. Statistical analysis was performance by the Mann–Whitney U test. All results were considered statistically significant at *P* < 0.05. CRP, C-reactive protein; ESR, erythrocyte sedimentation rate; N/L ratio, neutrophil-to-lymphocyte ratio.

### Main risk factors of mortality in Mexican severe COVID-19 patients

**FIGURE 2:**
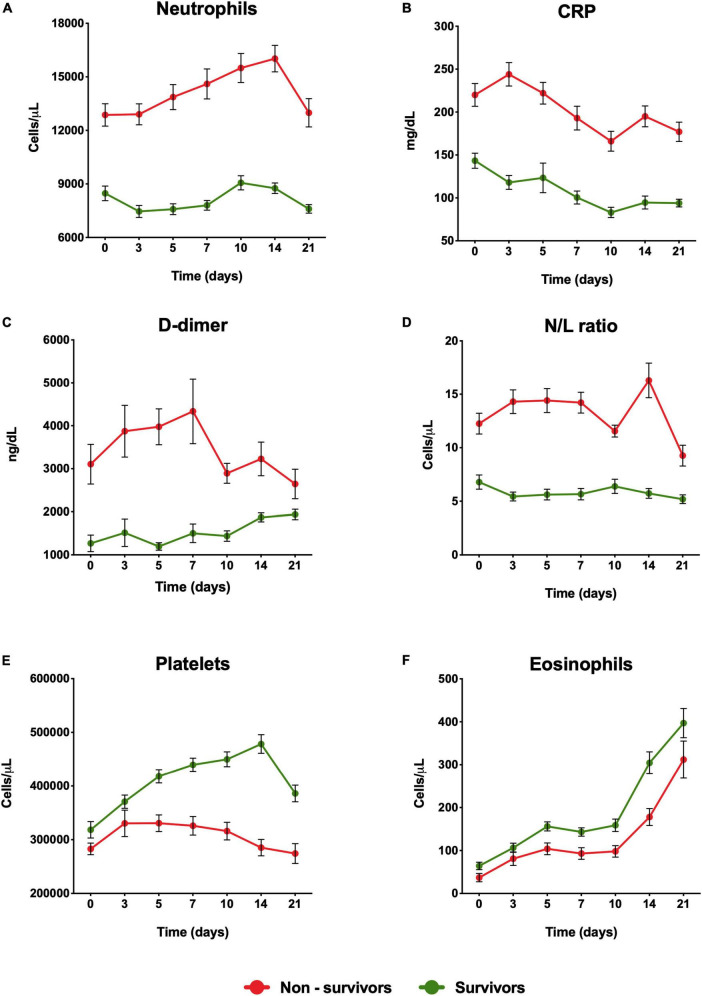
Dynamical changes in the levels of risk factors in severe COVID-19 patients during hospitalization. **(A)** Neutrophil, **(B)** CRP, **(C)** D-Dimer, **(D)** N/L ratio, **(E)** Platelets, **(F)** Eosinophils in patients within 21 days from admission onset. The patients were stratified into a non-survival group and a survival group. Data represents mean ± SD. CRP, C-reactive protein; N/L ratio, neutrophil-to-lymphocyte ratio.

The routine laboratory biomarkers were evaluated as far as 21 days. Our results showed that at baseline, the non-survivors group presented higher levels of neutrophils (12.9 ± 6.3 vs. 8.5 ± 4.9 × 10^3^ cells/μl, *P* < 0.0001), CRP (220 ± 133 vs. 143 ± 106 mg/ml, *P* < 0.0001), N/L ratio (12.3 ± 9.82 vs. 6.79 ± 8.02 *P* < 0.05) and D-dimer (3105 ± 4668 vs. 1267 ± 2347 ng/ml, *P* < 0.0001) and N/L ratio (12.3 ± 9.82 vs. 6.79 ± 8.02 P < 0.05) (Figures 2A–D) compared with survivor group. Meanwhile, the survivor group showed higher levels of platelets (318 ± 185 vs. 283 ± 109 × 10^3^ cells/μl, *P* = 0.015) and eosinophils (0.06 ± 0.10 vs. 0.04 ± 0.09 × 10^3^ cells/μl, *P* = 0.039) compared with the non-survivor group (Figures 2E,F). Dynamical changes in the levels of immune response in the non-survivor group showed that on day 3 of hospital stay, the highest level of CRP (244 ± 137 mg/ml) was observed (Figure 2B), at day 7 the highest concentration of D-dimer (4335 ± 7615 ng/ml) was reported (Figure 2C). Seven days later, the highest concentration was observed in the levels of neutrophils (16.0 ± 7.5 × 10^3^ cells/μl) (Figure 1A). On the contrary, in this group, it was observed that the lowest values for eosinophils (0.10 ± 0.13 × 10^3^ cells/μl) were at day 10 (Figure 2F) and platelets (285.3 ± 153.9 × 10^3^ cells/μl) at day 14 (Figure 2E). Over the period of analysis of the routine laboratory biomarkers, we evaluated the median of these variables throughout the follow-up of the participants. Results showed that the group of non-survivors showed an increase in the levels of leukocytes, neutrophils, CRP, D-dimer, ERS, and N/L ratio, and a decrease in the levels of eosinophil and platelets respect to the survivor group (Table 1).

**FIGURE 3:**
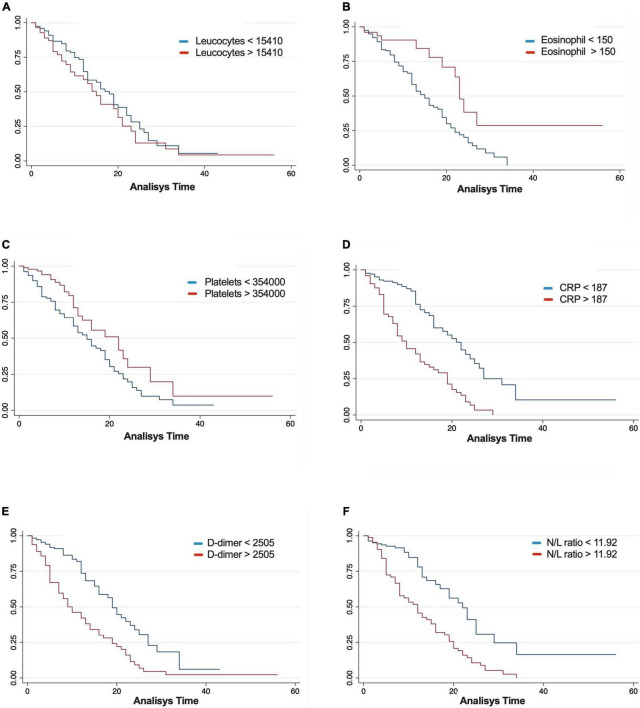
Kaplan–Meier survival estimates for leukocytes **(A)**, eosinophil **(B)**, platelets **(C)**, C-reactive protein (CRP) **(D)**, D-dimer **(E)**, and neutrophil-to-lymphocyte ratio (N/L ratio) **(F)**.

To evaluate the impact generated by the modifications in the levels of routine parameters, cut-off points were determined for the study population (Supplementary Table 3) with model unadjusted and adjusted by sex, age, and comorbidities to determine the mortality risk for each parameter. Our results showed that CRP (HR 3.04, CI 95% 2.03–4.56), N/L ratio (HR 2.68, CI 95% 1.78–4.05), and D-dimer (HR 2.46, CI 95% 1.67–3.64) were the highest predictive variables for mortality (*P* < 0.001), whereas the levels of eosinophils, lymphocytes and platelets showed an inverse association for mortality, as lower the level the higher risk for an adverse outcome (Table 2 and Figures 3A–F).

**TABLE 2:** Analysis of a survival-time model of the risk factors associated with mortality in severe COVID-19 patients.

	HR [CI 95%]	*P*-value
Leukocytes (×10^3^ cells/μL)	1.31 [0.87, 1.98]	0.19
Neutrophils (×10^3^ cells/μL)	2.47 [1.64, 3.72]	< 0.001
Lymphocytes (×10^3^ cells/μL)	0.49 [0.33, 0.72]	< 0.001
Eosinophil (×10^3^ cells/μL)	0.40 [0.22, 0.75]	0.004
Platelets (×10^3^ cells/μL)	0.56 [0.35, 0.89]	0.010
CRP (mg/mL)	3.04 [2.03, 4.56]	< 0.001
D-dimer (ng/mL)	2.46 [1.67, 3.64]	< 0.001
ESR (mm/h)	1.13 [0.76, 1.68]	0.52
N/L ratio	2.68 [1.78, 4.05]	< 0.001

Data are presented as median and 95% confidence interval [CI 95%]. All results were considered statistically significant at *P* < 0.05. CRP, C-reactive protein; ESR, Erythrocyte sedimentation rate, N/L ratio, neutrophil-to-lymphocyte ratio.

Interestedly, inflammatory markers such as neutrophils, CRP, D-dimer, and N/L ratio considerably increased the risk of death (Table 2). On the other hand, the levels of lymphocytes, eosinophils, and platelets were associated with improving the survival of the patients (Table 2). After adjusting the analysis by covariates (age, sex, and comorbidities), leukocytes (HR 0.9996 [0.99, 0.99], *P* = 0.03) and neutrophils (HR 1.0003 [CI 95% 1.00, 1.00], *P* = 0.007) showed a significant association with mortality (Table 3).

**TABLE 3:** Analysis of risk factors associated with mortality in severe COVID-19 patients by adjusted model.

Characteristic	HR [CI 95%]	*P*-value
Leukocytes (×10^3^ cells/μl)	0.9996 [0.99–0.99]	0.030
Neutrophils (×10^3^ cells/μl)	1.0003 [1.00–1.00]	0.007

Statistical analysis was performance by random-effects parametric survival-time model with adjusted by sex, age, and comorbidities. Log likelihood = −180.82; *P* < 0.0001. All results were considered statistically significant at *P* < 0.05.

## Discussion

In this study, the baseline, and subsequent changes of the routine laboratory biomarkers among the non−survival group were characterized by significantly higher levels of leukocytes, neutrophil count, N/L ratio, and inflammatory as well as coagulation markers such as CRP and D-dimer, while lymphocytes, eosinophils, and platelets count were significantly decreased. Besides, after estimating cut-off values, it was found that CRP, N/L ratio, neutrophils, and D-dimer are better predictors of mortality among the population. Our results strengthened the association between inflammatory response and mortality in patients with severe COVID-19. The proinflammatory biomarkers related to the Th-1 pathway such as leukocytes, neutrophils, CRP, and D-dimer were significantly associated with mortality in patients with severe COVID-19. During COVID-19 disease, there is a dysregulation of the inflammatory immune response and an increase of proinflammatory marker production. This alteration results in a dynamic process associated with tissue damage and multiple organ failure (17). Some studies identify the role of proinflammatory markers such as CRP, interleukine-6, and neutrophil count in the prediction of a worse prognosis in patients with COVID-19. Our results were accordingly with these findings, and introduce the concept of dynamic change in biomarkers as another factor associated with severity, whereas patients with mild disease are characterized by the presence of anti-inflammatory, prophagocytic, and antigen-presenting macrophages in the lungs, severe, and critical COVID-19 leads to an enrichment of hyperinflammatory macrophages with aberrant response in cytokines and inflammatory gene expressions (18). Previous reports highlighted that the increase of serum cytokines was related with an unbalance ratio with a decrease in lymphocytes and an increase neutrophils levels among patients with severe COVID-19 (19). This is consistent with previous studies, which showed that an excessively elevated neutrophil count released neutrophil DNA, and associated proteins that led to tissue damage, severe pneumonia, and death (19). High CRP levels are also linked to severe shock, systemic inflammatory response syndrome, and multiple organ dysfunction syndrome (20). In fact, a previous study showed that the cutoff point of CRP (≥40 mg/L) performed well in predicting mortality in patients with COVID-19 (21). Thus, the dynamic profile of CRP, has clinical relevance due to our results showed that CRP (>187 mg/L) increased 3.04 times the mortality risk in the population analyzed. This strengthened the suggestion of monitoring levels of CRP in these patients to identify a timely effective treatment to improve the prognosis of COVID−19. The latter was confirmed in hospitalized patients with hypoxia (SpO2 < 92%) in whom the addition of tocilizumab reduced the mortality rate by 15% at 28 days of follow-up whenever they had evidence of systemic inflammation (CRP ≥ 75mg/L) (22).

Another factor that contributes to the poor prognosis of the pathology is D-dimer, our results showed that D-dimer is a predictive variables for mortality. This is according with other studies that has been show similar results. In fact, a study reported that D-dimer levels increased in 36–43% of COVID-19 patients (23). Other study in the Turkish population showed that not only was D-dimer elevation common among patients diagnosed with COVID-19, but that this increase was associated with disease severity and mortality (24). A recent systematic review concluded that D-dimer is an independent predictor of COVID-19 mortality, even after subgroup analysis (countries, sample size, study design) (25). Mortality in COVID-19 patients has also been associated with changes in lymphocytes and platelets. In fact, it has been reported that in non-survivors, there is a decrease in levels of both parameters (26). Eosinophils are less well characterized in relation to COVID-19, thus some reports have found an association with the severity of the disease (27). In this sense, we found an inverse association of eosinophil levels with mortality. Lower levels of eosinophils might facilitate an alternative route of hyperinflammation, mainly due to Th-1. Additionally, the severity of the immuno-inflammatory state and dysregulation of the immune response are also related to alterations in levels of neutrophils and lymphocytes, which results in an increase in the N/L ratio. However, little number of studies consider the cost-benefit of this type of marker as an early indicator of severity in COVID-19. A meta-analysis showed that increased N/L ratio level have been associated with enhanced inflammation and a poor prognosis in COVID-19 patients, which is similar to our results (28).

This is the first evidence of the impact of routine laboratory biomarkers for mortality prediction in Mexican population. Because the Mexican health system continues to face challenges and has limited resources, the use of these routine laboratory biomarkers, particularly the N/L ratio and absolute neutrophil levels, and possibly special attention to eosinophils, could have an impact on the treatment of COVID-19 patients.

Our study has some limitations; firstly, only patients admitted to the hospital with severe COVID-19 were included, which leads to selection bias and therefore limits the overall applicability of the results. Besides, we were not able to measure specific molecules such as cytokines to reinforce our findings. However, to reduce the bias toward having heterogeneous disease conditions, critical illness cases (patients who required non-invasive or invasive mechanical ventilation) were not included in our study.

## Conclusion

The prompt dynamic analysis of biomarkers should be used for the early and appropriate detection of risk in patients with COVID-19 and making interventions accordingly. Our results suggest that leukocytes, neutrophil count, N/L ratio, CRP, and D-dimer values predicted mortality in patients with severe COVID-19 admitted to our hospital. These results may be used in the development of early strategies, which can also assist in the better management of patients.

## Data availability statement

The original contributions presented in this study are included in the article/Supplementary material, further inquiries can be directed to the corresponding author.

## Ethics statement

This study was approved by the Ethics Committee of the Hospital Regional de Alta Especialidad de la Península de Yucatán (2020-023), and it was conducted in accordance with the Declaration of Helsinki.

## Author contributions

AC-T, VÁ-S, and AA-N designed the study and wrote the manuscript. AC-T, VÁ-S, and RL performed the experiments. AC-T, AG-G, and AA-N analyzed the data. All authors contributed to the manuscript.
