# Supplementary material for: Dynamic inflammatory response among routine laboratory biomarkers and their predictive ability for mortality in patients with severe COVID-19
Source: Front Med (Lausanne). 2022 Nov 17;9:1047304. doi: 10.3389/fmed.2022.1047304 (PMC9714476; doi:10.3389/fmed.2022.1047304)
Supplement: Supplementary file 1 [file Table_1.DOCX]

**Supplementary Table 1.** General and clinical characteristics of severe COVID-19 patients (n=250).

| **Characteristics** |  |
| --- | --- |
| Age (years) | 54.3 ± 15.3 |
| **Sex, n (%)** |  |
| Male | 170 (68.0) |
| Female | 80 (32.0) |
| **Comorbidities, n (%)** |  |
| Obesity | 129 (51.6) |
| HBP | 83 (33.2) |
| T2D | 76 (30.4) |
| Smoking | 19 (7.6) |
| Cardiopathy | 13 (5.2) |
| CKD | 9 (3.6) |
| Dyslipidemia | 7 (2.8) |
| COPD | 7 (2.8) |
| Hepatopathy | 5 (2.0) |
| HIV | 5 (2.0) |
| Period of hospitalization (days) | 9.42 ± 8.23 |
| O^2^ saturation | 81.4 ± 15.3 |
| Eosinophil (absolute count) | 53.1 ± 103 |
| CRP (mg/L) | 175 ± 124 |
| D-dimer (ng/mL) | 2010 ± 3584 |
| N/L ratio | 11.9 ± 10.8 |

Values are present as mean ± SD, frequencies and percentages. HBP: High Blood Pressure; T2D: Type 2 Diabetes; COPD**:** Chronic Obstructive Pulmonary Disease; CKD: chronic kidney disease; HIV: human immunodeficiency virus. O_2_ saturation**:** oxygen saturation; AEC: Absolute Eosinophiles Count; CRP: C-reactive protein; neutrophil-to-lymphocyte ratio.

**Supplementary Table 2.** Comparative analysis of characteristics and comorbidities of the population according to survival status (n = 250).

| **Characteristic**  n (%) | **Survivors**  n = 148 | **Non-survivors**  n = 102 | **P value** |
| --- | --- | --- | --- |
| **Sex** |  |  |  |
| Men | 97 (65) | 73 (71) | 0.31 |
| Women | 51 (34) | 29 (28) |  |
| HBP | 44 (29) | 39 (38) | 0.16 |
| T2D | 38 (25) | 38 (37) | 0.50 |
| Obesity | 74 (50) | 55 (54) | 0.54 |
| COPD | 4 (2.0) | 3 (3.0) | 0.91 |
| Smoking | 11 (7.0) | 8 (8.0) | 0.94 |
| Cardiopathy | 8 (5.0) | 5 (5.0) | 0.86 |
| CRI | 4 (2.0) | 5 (5.0) | 0.35 |
| Hepatopathy | 2 (1.0) | 3 (3.0) | 0.37 |
| HIV | 3 (2.0) | 2 (2.0) | 0.91 |
| Dyslipidemia | 3 (2.0) | 4 (4.0) | 0.37 |
| Mobility | 109 (73) | 77 (75) | 0.74 |

Data are presented by frequency and percentages. HBP: High Blood Pressure; T2D: Type 2 Diabetes; COPD**:** Chronic Obstructive Pulmonary Disease; CRI: chronic renal insufficiency; HIV: human immunodeficiency virus. Statistical analysis was performance by chi-squared test. All results were considered statistically significant at P < 0.05.

**Supplementary Table 3.** Diagnostic performances of risk of routine biochemical parameters.

|  | **Cut Off** | **Sensibility** | **Specificity** | **Precision** |  |
| --- | --- | --- | --- | --- | --- |
| Leukocytes (x 10^3^ cells/µL) | | > 15.4 | 0.35 | 0.85 | 0.65 |
| Neutrophils (x 10^3^ cells /µL) | | > 10.8 | 0.63 | 0.83 | 0.75 |
| Lymphocytes (x 10^3^ cells/µL) | | > 0.83 | 0.53 | 0.16 | 0.32 |
| Eosinophil (x 10^3^ cells/µL) | | > 0.15 | 0.13 | 0.73 | 0.48 |
| Platelets (x 10^3^ cells/µL) | | < 354 | 0.27 | 0.54 | 0.43 |
| CRP (mg/mL) | | >187 | 0.55 | 0.87 | 0.74 |
| D-dimer (ng/mL) | | > 2505 | 0.50 | 0.92 | 0.75 |
| ERS (mm/h) | | > 31 | 0.47 | 0.72 | 0.60 |
| N/L ratio | | > 11.9 | 0.65 | 0.87 | 0.78 |

CRP: C-reactive protein; ESR: Erythrocyte sedimentation rate, N/L ratio: neutrophil-to-lymphocyte ratio.
